# P01 Safety of continuous IV antibiotic infusions via peripheral cannulas in home hospital settings: a retrospective 2 year audit

**DOI:** 10.1093/jacamr/dlaf239.005

**Published:** 2026-01-05

**Authors:** Mathew Sermanni, Martin Hewitt, Jared Eisemann

**Affiliations:** Hospital in the Home, Mater Hospital Brisbane, Brisbane, QLD, Australia; Hospital in the Home, Mater Hospital Brisbane, Brisbane, QLD, Australia; Hospital in the Home, Mater Hospital Brisbane, Brisbane, QLD, Australia

## Abstract

**Background:**

For select patient groups, IV antibiotic therapy at home is both safe and reduces cost/inpatient burden. Vascular Access Devices represent a significant cost to healthcare systems. For Hospital in the Home (HITH) patients requiring short durations of IV Abx, PIVCs are cheaper and less invasive than PICCs. However, there is a lack of evidence on safety outcomes of continuous antibiotic infusions delivered by PIVCs in HITH setting.

**Objectives:**

Retrospective analysis using the Mater Hospital Brisbane’s HITH database aimed to demonstrate that delivering continuous antibiotic infusions via PIVCs are safe in HITH settings, and to guide prospective study into safety outcomes/complication rates in this patient cohort.

**Methods:**

All patients admitted between April 2022–April 2024 were filtered by both vascular access device type and antibiotic prescribed. Electronic scanned records of admission were reviewed for safety outcomes.

**Results:**

A total of 126 patients were identified who received continuous antibiotic therapy for a mean duration of 3.24 days. A total of 167 PIVCs were inserted into our patient cohort, for a combined total of 408 days of PIVC dwell time. There were no significant PIVC-related complications (thrombophlebitis, blood stream infection, readmission due to PIVC-related treatment failure) and 29 minor PIVC-related complications (occlusion/infiltration, dislodgement, discomfort, phlebitis).

**Conclusions:**

Continuous antibiotic infusions via PIVC in HITH settings appear to be safe. We observed an approximate 17% PIVC failure rate in our cohort, with no significant PIVC-related complications. Published literature suggests overall PIVC failure rate lies between 35-50%. Future study into safety outcomes of HITH patients who receive continuous antibiotic infusions via PIVC (versus PICC?) is warranted.

